# Analysis of Physicians’ Probability Estimates of a Medical Outcome Based on a Sequence of Events

**DOI:** 10.1001/jamanetworkopen.2022.18804

**Published:** 2022-06-27

**Authors:** Hal R. Arkes, Scott K. Aberegg, Kevin A. Arpin

**Affiliations:** 1 Harding Center for Risk Literacy, University of Potsdam, Potsdam, Germany; 2 Department of Psychology, The Ohio State University, Cleveland Heights; 3 Department of Internal Medicine, Pulmonary Division, University of Utah, Salt Lake City; 4 Coventry, Connecticut

## Abstract

**Importance:**

The probability of a conjunction of 2 independent events is the product of the probabilities of the 2 components and therefore cannot exceed the probability of either component; violation of this basic law is called the conjunction fallacy. A common medical decision-making scenario involves estimating the probability of a final outcome resulting from a sequence of independent events; however, little is known about physicians’ ability to accurately estimate the overall probability of success in these situations.

**Objective:**

To ascertain whether physicians are able to correctly estimate the overall probability of a medical outcome resulting from 2 independent events.

**Design, Setting, and Participants:**

This survey study consisted of 3 separate substudies, in which 215 physicians were asked via internet-based survey to estimate the probability of success of each of 2 components of a diagnostic or prognostic sequence as well as the overall probability of success of the 2-step sequence. Substudy 1 was performed from April 2 to 4, 2021, substudy 2 from November 2 to 11, 2021, and substudy 3 from May 13 to 19, 2021. All physicians were board certified or board eligible in the primary specialty germane to the substudy (ie, obstetrics and gynecology for substudies 1 and 3 and pulmonology for substudy 2), were recruited from a commercial survey service, and volunteered to participate in the study.

**Exposures:**

Case scenarios presented in an online survey.

**Main Outcomes and Measures:**

Respondents were asked to provide their demographic information in addition to 3 probability estimates. The first substudy included a scenario describing a brow presentation discovered during labor; the 2 conjuncts were the probabilities that the brow presentation would resolve and that the delivery would be vaginal. The second substudy involved a diagnostic evaluation of an incidentally discovered pulmonary nodule; the 2 conjuncts were the probabilities that the patient had a malignant condition and that a technically successful transthoracic needle biopsy would reveal a malignant condition. The third substudy included a modification of the first substudy in an attempt to debias the conjunction fallacy prevalent in the first substudy. Respondents’ own probability estimates of the individual events were used to calculate the mathematically correct conjunctive probability.

**Results:**

Among 215 respondents, the mean (SD) age was 54.0 (9.5) years; 142 respondents (66.0%) were male. Data on race and ethnicity were not collected. A total of 168 physicians (78.1%) estimated the probability of the 2-step sequence to be greater than the probability of at least 1 of the 2 component events. Compared with the product of their 2 estimated components, respondents overestimated the combined probability by 12.8% (95% CI, 9.6%-16.1%; *P* < .001) in substudy 1, 19.8% (95% CI, 16.6%-23.0%; *P* < .001) in substudy 2, and 18.0% (95% CI, 13.4%-22.5%; *P* < .001) in substudy 3, results that were mathematically incoherent (ie, formally illogical and mathematically incorrect).

**Conclusions and Relevance:**

In this survey study of 215 physicians, respondents consistently overestimated the combined probability of 2 events compared with the probability calculated from their own estimates of the individual events. This biased estimation, consistent with the conjunction fallacy, may have substantial implications for diagnostic and prognostic decision-making.

## Key Points

**Question:**

Are physicians able to accurately estimate the overall probability of a medical outcome resulting from 2 independent events?

**Findings:**

In this survey study of 215 physicians, most respondents (78.1%) estimated the probability of a medical outcome resulting from a 2-step sequence to be greater than the probability of at least 1 of the 2 component events, a result that was mathematically incoherent (ie, formally illogical and mathematically incorrect).

**Meaning:**

This study’s findings suggest that because many diagnostic and prognostic decisions require more than 1 step or the consideration of more than 1 probability, misestimation of the overall probability of success when 2 or more independent events are involved (termed the conjunction fallacy) is likely to be a source of diagnostic and prognostic error.

## Teaser

This survey study assesses whether physicians are able to correctly estimate the overall probability of a medical outcome resulting from 2 independent events.

## Introduction

In 3 published studies,^1,2,3^ physicians or medical students judged the probability of having both a typical symptom and an atypical symptom of a disease to be more probable than having only the atypical symptom of that disease. Such judgments are mathematically incoherent (ie, formally illogical and mathematically incorrect). The probability of a conjunction of 2 independent events is the product of the probabilities of the 2 components and therefore cannot exceed the probability of either component. A violation of this basic law of probability is called the conjunction fallacy.^1^

A more common situation in which the conjunction fallacy might arise in the medical setting occurs when physicians are required to estimate the likelihood of an outcome that requires 2 or more component probabilities. For example, if the successful outcomes of both step A and step B are necessary for an overall procedure to be successful, then the successful occurrence of the overall 2-step procedure cannot exceed the probability of either component. Because many diagnostic and prognostic decisions require more than 1 step, or the consideration of more than 1 probability, physicians must consider the conjunction of these multiple steps or probabilities to render an accurate assessment of the overall outcome. In fact, the impetus for this research project was a real case involving a medical tragedy. In that case, patient counseling related to probabilities was misguided in a way consistent with the conjunction fallacy, resulting in the loss of a child due to injuries sustained during prolonged labor.

This survey study aimed to ascertain whether physicians were able to correctly estimate the overall probability of a medical outcome resulting from 2 independent events. The hypothesis was that physicians would estimate the probability of a 2-step conjunction to be greater than the probability of 1 or both of its 2 components.

## Methods

This survey study comprised 3 substudies. Substudy 1 was performed from April 2 to 4, 2021, substudy 2 from November 2 to 11, 2021, and substudy 3 from May 13 to 19, 2021. Respondents provided electronic informed consent before being permitted access to the internet survey material. All 3 substudies were deemed exempt by the institutional review board of The Ohio State University. This study followed the Checklist for Reporting Results of internet E-Surveys (CHERRIES)^4^ reporting guideline for web-based survey studies (eMethods 4 in the Supplement).

We used 3 surveys (1 survey in each of 3 substudies) to evaluate the commission of the conjunction fallacy by survey respondents. We conducted 2 substudies to assess the presence of the conjunction fallacy in an obstetric context and a third substudy to examine its presence in a pulmonary context. Using internet-based surveys, physicians were presented with a scenario involving a sequence of events related to their medical specialty. Respondents were asked to judge the overall probability of the conjunction and the probability of the individual conjuncts.

**Figure 1.**
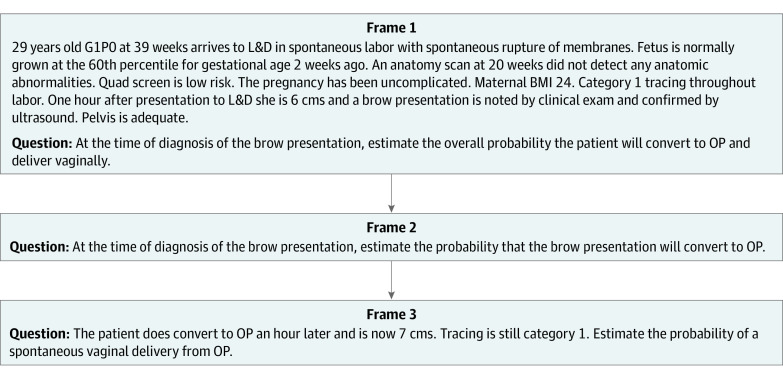
Successive Frames of the Scenario Involving a Brow Presentation During Labor Frames from substudy 1, excluding frames related to qualifying and demographic information. BMI indicates body mass index; G1PO, gravida 1 para 0 (1 pregnancy and no live births); L&D, labor and delivery; and OP, occiput posterior.

The first substudy described a scenario in which brow presentation was discovered during labor (Figure 1). To assess the overall probability of a successful vaginal delivery, an obstetrician must consider the probability of the brow presentation converting to a deliverable position and the probability of delivering vaginally from that converted position. This substudy used occiput posterior as the deliverable position.

**Figure 2.**
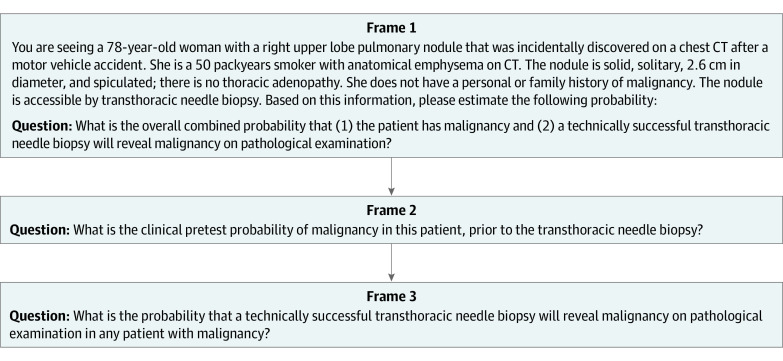
Successive Frames of the Scenario Involving a Pulmonary Nodule Frames from substudy 2, excluding frames related to qualifying and demographic information. CT indicates computed tomography.

The second substudy involved the diagnostic evaluation of an incidentally discovered pulmonary nodule (Figure 2). The probability that a biopsy reveals cancer in the patient is a function of both the probability that the nodule is cancerous and the probability of the biopsy successfully detecting cancer in the presence of a cancerous nodule.

The third substudy included a modification of the first substudy in an attempt to debias the physicians’ manifestation of the conjunction fallacy prevalent in the first substudy. Research pertaining to judgment and decision-making^5,6^ and problem solving^7^ has found that decomposing a task into its components substantially improves performance. However, none of these studies^5,6,7^ attempted to address the conjunction fallacy. Our debiasing survey required respondents to consider the conjunction’s components before estimating the overall probability. This approach contrasted with that of our first 2 substudies, in which we asked for the 2 components’ probability estimates after the overall probability estimate was rendered. Thus, if any physician had realized the component probabilities should have been considered when contemplating the previous overall estimate, it was too late to rectify the overall estimate because respondents were not allowed to change their previous responses as they progressed through the successive frames of the survey. We hypothesized that the modified approach used in substudy 3 would improve physicians’ estimation of the overall probability of the 2-step sequence.

### Recruitment of Participants

Independent samples of physicians were recruited for participation in the 3 substudies. Reckner Healthcare,^8^ a commercial survey service, was used to recruit physicians who were paid an honorarium for participation. Reckner Healthcare maintains panels of physicians in several specialties. To qualify for participation in our survey study, physicians had to be board certified or board eligible in the primary specialty germane to one of the substudies (ie, obstetrics and gynecology for substudies 1 and 3 and pulmonology for substudy 2). Data from 1 physician were not collected in substudy 1 because that person did not attend deliveries. Respondents were asked to provide basic demographic information in addition to their 3 probability estimates (2 component probabilities and 1 combined probability). Data on race or ethnicity were not collected from respondents because we did not think that the cognitive processes responsible for manifestation of the conjunction fallacy would vary depending on racial and ethnic characteristics.

Response rates for all surveys were high because all participants had previously volunteered to participate in the Reckner Healthcare panel of survey respondents. Of 242 eligible physicians who accessed the survey websites, 215 provided data, for a response rate of 89%. Further information on the response rate is available in eMethods 3 in the Supplement.

### Probability Estimates

The first type of fallacy assessed was the single conjunction fallacy, defined as estimation of the probability of the conjunction that was greater than or equal to 1 of the components. If the probability estimate of the conjunction was equal to one of the components, it was counted as a single conjunction fallacy only if the probability of the other component was judged to be less than 1.0. The second type of fallacy assessed was the double conjunction fallacy, defined as estimation of the probability of the conjunction that was greater than both components. Additional details about these definitions are available in eMethods 1 in the Supplement.

### Statistical Analysis

We intended for the sample size for each of our 3 substudies to approximate the mean of the sample size (N = 72) of the 2 previous conjunction fallacy studies^1,3^ that surveyed physicians rather than medical students. Owing to an administrative miscommunication, our second substudy (pulmonary nodule) had a slightly larger sample than the other 2 substudies. We used 2-tailed *P* < .05 as our definition of statistical significance. Cohen *d* was used for all comparisons to measure the effect size, with *d* > 0.80 considered a large effect size.^9^ We used IBM SPSS software, version 27 (IBM Corp), for all statistical analyses.

Dependent *t* tests were used for all within-group comparisons, such as comparisons of the product of the 2 constituent probabilities’ divergence from a physician’s estimated conjunction probability. The χ^2^ test was used to compare the frequency of conjunction fallacies in substudies 1 and 3. Pearson correlation coefficients were used to examine the association between demographic factors and dependent variables. For the time taken to complete each survey, we used medians and IQRs because of the nonnormality of that distribution.

The hyperlinks to the preregistration websites of the 3 substudies are available in eMethods 2 in the Supplement. Original data are available by contacting the corresponding author.

## Results

### Participant Characteristics

Among 215 physicians, 142 (66.0%) were male, and 73 (34.0%) were female, with a mean (SD) age of 53.6 (9.5) years; the mean (SD) time since obtaining a medical degree was 27.5 (10.6) years. As discussed in the Methods section, data on race and ethnicity were not collected. A total of 67 obstetricians participated in the brow presentation survey, 84 pulmonologists participated in the pulmonary nodule survey, and 64 obstetricians participated in the debiasing survey. The mean (SD) age of respondents (54.8 [9.0] years in substudy 1, 53.3 [9.6] years in substudy 2, and 52.9 [9.8] years in substudy 3) and the mean (SD) time since obtaining a medical degree (29.0 [11.3] years in substudy 1, 27.4 [10.3] years in substudy 2, and 25.9 [10.4] years in substudy 3) were similar among groups. However, substudy 2 had a higher proportion of male physicians (71 pulmonologist [84.5%]) compared with substudy 1 (43 obstetricians [64.2%]) and substudy 3 (28 obstetricians [43.8%]) (Table 1). Survey completion time was also slightly higher in substudy 2 (median [IQR], 2.6 [1.3-2.4] minutes) vs substudy 1 (median [IQR], 2.1 [1.6-3.1] minutes) and substudy 3 (median [IQR], 2.0 [1.5-3.0] minutes). Overall, 168 physicians (78.1%) committed the conjunction fallacy (Table 2).

**Table 1.** Demographic Characteristics of Respondents and Median Completion Time of Survey

Characteristic	Brow presentation	Pulmonary nodule	Debiasing the brow presentation
Total respondents, No.	67	84	64
Time since medical degree, mean (SD), y	29.0 (11.3)	27.4 (10.3)^a^	25.9 (10.4)
Age, mean (SD), y	54.8 (9.0)	53.3 (9.6)	52.9 (9.8)^a^
Sex, No. (%)			
Female	24 (35.8)	13 (15.5)	36 (56.2)
Male	43 (64.2)	71 (84.5)	28 (43.8)
Survey completion time, median (IQR), min	2.1 (1.6-3.1)	2.6 (1.3-2.4)	2.0 (1.5-3.0)

^a^ Two physicians did not provide data for this item.

**Table 2.** Summary of Results From All 3 Substudies

Response	Participants, No./total No. (%)
Substudy 1: brow presentation	
Single conjunction fallacy	46/67 (68.7)
Double conjunction fallacy	4/67 (6.0)
Total conjunction fallacy errors	50/67 (74.6)
Substudy 2: pulmonary nodule	
Single conjunction fallacy	54/84 (64.3)
Double conjunction fallacy	19/84 (22.6)
Total conjunction fallacy errors	73/84 (86.9)
Substudy 3: debiasing the brow presentation	
Single conjunction fallacy	42/64 (65.6)
Double conjunction fallacy	3/64 (4.7)
Total conjunction fallacy errors	45/64 (70.3)

### Brow Presentation During Labor

In substudy 1, 50 of 67 obstetricians (74.6%) committed the conjunction fallacy, with 46 committing the single conjunction fallacy and 4 committing the double conjunction fallacy (Table 2). Compared with the product of their 2 estimated components, respondents overestimated the combined probability by 12.8% (95% CI, 9.6%-16.1%), which was mathematically incoherent (reported deviations always represent absolute differences on the probability scale). This deviation was statistically significant (*t*_66_ = 7.94; *P* < .001; Cohen *d* = 0.97 [95% CI, 0.68-1.26]) (eFigure 1 in the Supplement).

### Pulmonary Nodule

In substudy 2, 73 of 84 pulmonologists (86.9%) committed the conjunction fallacy, with 54 committing the single conjunction fallacy and 19 committing the double conjunction fallacy (Table 2). Compared with the product of their 2 estimated components, respondents overestimated the combined probability by 19.8% (95% CI, 16.6%-23.0%), which was mathematically incoherent. This deviation was statistically significant (*t*_83_ = 12.30; *P* < .001; Cohen *d* = 1.34 [95% CI, 1.04-1.64]) (eFigure 2 in the Supplement).

### Debiasing Brow Presentation During Labor

In substudy 3, 45 of 64 obstetricians (70.3%) committed the conjunction fallacy, with 42 committing the single conjunction fallacy and 3 committing the double conjunction fallacy (Table 2). Compared with the product of their 2 estimated components, respondents overestimated the combined probability by 18.0% (95% CI, 13.4%-22.5%), which was mathematically incoherent. This deviation was statistically significant (*t*_63_ = 7.89; *P* < .001; Cohen *d* = 0.99 [95% CI, 0.68-1.28]) (eFigure 3 in the Supplement).

We compared the results of this debiasing substudy with the results of substudy 1, in which the overall probability was solicited first. Results of the χ^2^ test in which the 2 possible results were manifestations of either type of conjunction fallacy vs not manifestations of either type of conjunction fallacy were not statistically significant when we compared the 2 studies (χ^2^_1_ = 0.128; *P* = .72 [Yates correction]). Additional analyses of the frequency of the single conjunction fallacy are available in eAppendix 2 in the Supplement.

We examined whether male and female physicians had different levels of overestimation. Findings from these *t* tests were not significant in any of the 3 substudies (substudy 1: *t* = 0.85 [*P* = .40]; substudy 2: *t* = 1.58 [*P* = .12]; substudy 3: *t* = 0.68 [*P* = .53]). We also examined whether the time since obtaining a medical degree was correlated with overestimation magnitude. This correlation was not significant in any of the 3 substudies (substudy 1: *r* = 0.239 [*P* = .05]; substudy 2: *r* = 0.074 [*P* = .51]; substudy 3: *r* = −0.098 [*P* = .44]).

**Figure 3.**
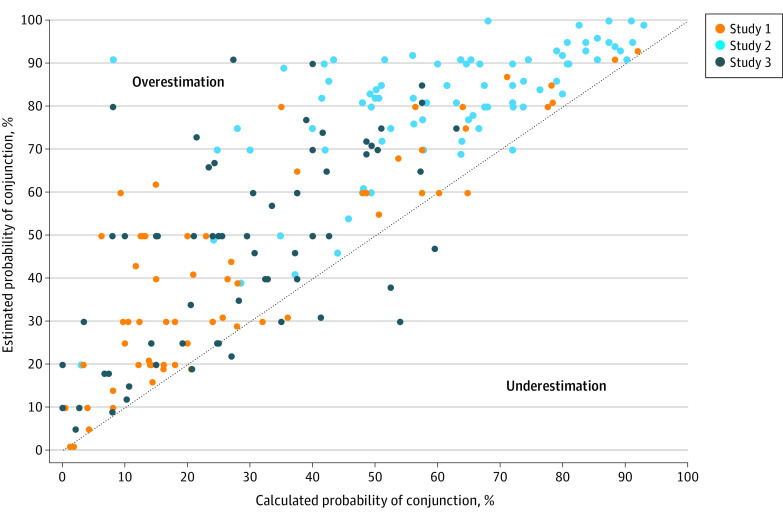
Comparison of All Respondents' Estimation of the Conjunction Probability and the Calculated Conjunction Probability Data above the dotted line represent an overestimation of the conjunction probability. The calculated conjunction probability was obtained using respondents’ individual component probability estimations. Substudy 1 included a scenario describing a brow presentation occurring during labor, substudy 2 involved diagnostic evaluation of an incidentally discovered pulmonary nodule, and substudy 3 included a modification of substudy 1 in an attempt to debias the conjunction fallacy prevalent in substudy 1.

With regard to the 2 component probability estimates in each of our 3 substudies, the ranges of these 6 estimates were wide (Figure 3). The ranges for each of the 2 probability estimates in the initial brow presentation scenario were 2% to 93% and 15% to 100%, respectively. In the pulmonary nodule scenario, ranges were 19% to 100% and 15% to 100%, respectively. In the debiasing substudy, ranges were 0% to 93% and 0% to 94%, respectively. The ranges of the conjunction estimates were also large, with substudy 1 ranging from 1% to 93%, substudy 2 ranging from 20% to 100%, and substudy 3 ranging from 5% to 91%.

## Discussion

The findings of this survey study suggest that physician misestimation of the probability of medical outcomes may be common. Estimating the successful outcome of a multistep procedure is a common task among physicians. If this task is performed in a logically flawed manner, overall estimates will be inaccurate. Across our 3 substudies, 78.1% of physicians committed the conjunction fallacy. This behavior was associated with substantial overestimation of the probability of the 2-step sequences. Overestimation has the potential to reduce the quality of medical care in any of the myriad scenarios in which decisions depend on probability estimates.^10^

We used each respondent’s component probability estimates to calculate the normative conjunction probability estimate. Therefore, any incorrect estimation of the conjunction could not be associated with misestimation of the true value of either component. The wide range of conjunction probability estimates suggests that a large proportion of the physicians’ estimates were incorrect. Such high variability disconfirms the hypothesis that physicians would correctly estimate the conjunction directly despite incongruence of the conjunction with their estimates of the components. Previous research^11^ has found that additional experience with the events for which probabilities are to be estimated was not associated with reductions in overestimation. Thus, the considerable expertise and experience of the physicians in this study might not have proved helpful in this task.

Serious miscalculations of the conjunction were observed but not counted toward the frequency with which the conjunction fallacy was committed. For example, if a physician assigned probabilities of 0.70 to each component and 0.69 to the conjunction, this miscalculation was not counted as an example of a conjunction fallacy even though it overestimated the probability of both events occurring by 20% (69% vs 49%).

Our attempt to debias physicians’ probability estimates in our third substudy was not successful. Although judgment decomposition or disaggregation has had positive consequences in some judgment and problem-solving domains,^5,6,7^ that strategy was unable to ameliorate the manifestation of the conjunction fallacy in our first substudy. We required respondents to consider the probability of the components before soliciting their probability estimate of the 2-step sequence. Because the 2 component probabilities were clearly relevant to estimating the overall probability, we concluded that the physicians did not know (or did not recognize when to use) the multiplication rule for probability. Because our third substudy asked for the estimates of the components first and because these estimates were often provided in round numbers (eg, 30% or 40%), calculating the conjunctive estimate should have been relatively easy if physicians were aware of the multiplication rule. However, only 1 physician in these 3 substudies correctly estimated the conjunction probability to be exactly equal to the product of the components' estimates.

There have been many efforts to explain the manifestation of the conjunction fallacy. The earliest was representativeness, which was used to explain the initial double-symptom study.^1^ In such studies,^1,2^ many physicians, when first told the diagnosis of a patient, deemed the conjunction of an unusual symptom and a typical symptom to be more likely than the presence of only the unusual symptom. The conjunction seemed to be more representative of the diagnosis because, unlike the single unusual symptom scenario, the conjunction contained 1 symptom that was to be expected. In our opinion, representativeness did not apply in our substudies because there was no unusual component included in the 2-step sequence. In addition, previous studies^12,13,14^ have obtained results that contradicted the possibility of representativeness as an explanation.

A second possible explanation is based on a group of models that identified the reason for erroneous conjunctive estimates as the nonnormative manner in which the components’ probabilities were amalgamated into a conjunctive estimate. One of the most prominent is the configural weighted average model.^15^ According to this model, when 2 symptoms are the components of the conjunction, the estimator provides weights for both the probability of the less probable symptom and the most probable symptom and calculates the mean of these 2 products. Because the mean of 2 products must lie between the 2 components’ probabilities, double conjunction errors are inconsistent with this model. In our 3 substudies, approximately 12% of the physicians committed double conjunction errors. In one of the previous double-symptom studies using 2 surveys,^3^ 62% and 49% of the physicians committed double conjunction errors. Such data cast doubt on the configural weighted average model. Jenny et al^15^ discussed other models designed to explain the conjunction fallacy, but elaborating on each of these models is beyond the scope of the current article.

There are several caveats to keep in mind when considering the applicability of these models. First, there is evidence that substantial heterogeneity exists in the strategies different people use to arrive at a conjunctive estimate.^11,15^ Thus, no single model is likely to explain the behavior of all conjunctive estimators. Second, to our knowledge, no previous study has used a multistep sequence to assess the presence of the conjunction fallacy. This omission is important because some models and explanations seem less applicable in the multistep context compared with the traditional double-symptom context. Because all components of a sequence must occur for the sequence to be successful, the similarity of each component to the conjunction, which is the most important facet of the representativeness heuristic, simply does not apply.

A more general explanation for our results is based on the finding that many physicians may not be facile in the calculation of probability or even basic numeracy.^16,17,18,19,20,21^ For example, Eddy^17^ provided base rate, sensitivity, and other information that should have enabled physicians to calculate the probability that a woman had breast cancer based on a positive result on mammography screening; 95% of physicians responded with a probability that was 10 times higher than the correct answer. Previous work^18,19,20,21^ summarizing numerous similarly concerning findings may lead one to conclude that physicians simply do not know the multiplicative rule for combining probabilities.

The medical consequences of the conjunction fallacy may be substantial. The normative approach for medical decision-making^22^ requires defining a threshold probability that warrants an action or intervention. In cases such as our obstetrics scenario, there is a threshold probability for successful vaginal delivery below which immediate cesarian delivery may be warranted to minimize fetal risk.^23^ Because the conjunction fallacy necessarily results in overestimation of the conjunction, commission of the fallacy could make it appear as though the threshold for immediate intervention has not been crossed when in reality it has (eAppendix 1 in the Supplement), potentially resulting in catastrophic maternal and/or fetal consequences, as in the case that inspired our research. In response to these issues, several researchers^20,21,24^ have recommended including greater emphasis on numeracy as well as statistical and probabilistic reasoning in medical education.

In the pulmonary nodule scenario, overestimation of the probability of both the presence of cancer and the ability to detect the cancer through biopsy may result in unnecessary confusion for both patient and physician. It may also lead to the selection of a suboptimal diagnostic strategy. Our respondents were not given alternative diagnostic choices, such as surgical resection. If they had been, comparing alternatives to select the best among them would not have been possible without proficiency in dealing with the probability of success of each strategy. If the success of a 2-step process were overestimated and then compared with a 1-step process, such as a surgical procedure, a rational comparison of the 2 options would not be possible.

### Limitations

This study has limitations. The boundary conditions of the conjunction fallacy are unknown, and more work will be required to delineate them. Although our findings were statistically significant and consistent across 3 samples of physicians, it remains unknown whether the results of written surveys are representative of physician choices when providing care to real patients.^25^

## Conclusions

In this survey study, most physicians (78.1%) committed the conjunction fallacy by estimating the probability of success of a 2-step sequence to be more likely than the probability of success of 1 or both of its constituent components. Such a pattern of probability estimation was mathematically incoherent, producing substantial overestimation of the probability of the conjunction. Because many diagnostic and prognostic decisions require more than 1 step or the consideration of more than 1 probability, this misestimation may have substantial implications for diagnostic and prognostic decision-making.

## Notes

Supplement. **eMethods 1.** Elaboration on the Definitions of Single and Double Conjunction Fallacies **eMethods 2.** URLs for Preregistration of Substudies **eMethods 3.** Response Rate Information **eMethods 4.** Survey Guidelines **eAppendix 1.** Analysis Comparing the Discrepancy Between the Conjunction Estimate and the Product of the 2 Components Among Physicians Who Committed Any Conjunction Fallacy vs Physicians Who Did Not **eAppendix 2.** Further Analysis of the Frequency of the Commission of the Single Conjunction Fallacy in the 3 Substudies **eFigure 1.** Probability Estimates From Participants in Substudy 1 (Brow Presentation During Labor) **eFigure 2.** Probability Estimates From Participants in Substudy 2 (Pulmonary Nodule) **eFigure 3.** Probability Estimates From Participants in Substudy 3 (Debiasing Brow Presentation During Labor)
eReferences
