# Supplementary material for: Analysis of Physicians’ Probability Estimates of a Medical Outcome Based on a Sequence of Events
Source: JAMA Netw Open. 2022 Jun 27;5(6):e2218804. doi: 10.1001/jamanetworkopen.2022.18804 (PMC9237793; doi:10.1001/jamanetworkopen.2022.18804)
Supplement: Supplement. — eMethods 1. Elaboration on the Definitions of Single and Double Conjunction Fallacies eMethods 2. URLs for Preregistration of Substudies eMethods 3. Response Rate Information eMethods 4. Survey Guidelines eAppendix 1. Analysis Comparing the Discrepancy Between the Conjunction Estimate and the Product of the 2 Components Among Physicians Who Committed Any Conjunction Fallacy vs Physicians Who Did Not eAppendix 2. Further Analysis of the Frequency of the Commission of the Single Conjunction Fallacy in the 3 Substudies eFigure 1. Probability Estimates From Participants in Substudy 1 (Brow Presentation During Labor) eFigure 2. Probability Estimates From Participants in Substudy 2 (Pulmonary Nodule) eFigure 3. Probability Estimates From Participants in Substudy 3 (Debiasing Brow Presentation During Labor) eReferences [file jamanetwopen-e2218804-s001.pdf]

Supplementary Online Content

Arkes HR, Aberegg SK, Arpin KA. Analysis of physicians' probability estimates of a medical outcome based on a sequence of events. *JAMA Netw Open*. 2022;5(6):e2218804. doi:10.1001/jamanetworkopen.2022.18804

eMethods 1. Elaboration on the Definitions of Single and Double Conjunction Fallacies

eMethods 2. URLs for Preregistration of Substudies

eMethods 3. Response Rate Information

eMethods 4. Survey Guidelines

eAppendix 1. Analysis Comparing the Discrepancy Between the Conjunction Estimate and the Product of the 2 Components Among Physicians Who Committed Any Conjunction Fallacy vs Physicians Who Did Not

eAppendix 2. Further Analysis of the Frequency of the Commission of the Single Conjunction Fallacy in the 3 Substudies

eFigure 1. Probability Estimates From Participants in Substudy 1 (Brow Presentation During Labor)

eFigure 2. Probability Estimates From Participants in Substudy 2 (Pulmonary Nodule)

eFigure 3. Probability Estimates From Participants in Substudy 3 (Debiasing Brow Presentation During Labor)

eReferences

This supplementary material has been provided by the authors to give readers additional information about their work.

© 2022 Arkes HR et al. *JAMA Network Open*.

eMethods 1. Elaboration on the Definitions of Single and Double Conjunction Fallacies

The following are the definitions of the 2 types of conjunction fallacies.

a. Estimating the probability of the conjunction to be greater than or equal to 1 of the components. Such estimates are called a "single conjunction fallacy." If the probability estimate of the conjunction was equal to one of the components, it counted as a single conjunction fallacy only if the probability of the other component was judged to be less than 1.0.
b. Estimating the probability of the conjunction to be greater than both components. This is called a "double conjunction fallacy."

With regard to the definition of a single conjunction fallacy we provide the following examples and our justifications.

Consider the following estimates provided by a physician where the first 2 numbers are the components' estimates and the third number is the estimate of the conjunction: 20, 50, 30. This is a typical single conjunction error, because the estimate of the conjunction is greater than the estimate of one of the components.

Now consider this trio of estimates: 30, 50, 30. The conjunction is not greater than either component. However, it is equal to the first component. This is logically impossible, and it would be scored as the commission of a single conjunction fallacy. If the estimate of the conjunction were equal to the estimate of the first component and the second component were 100%, then this pattern would be logically coherent, and it would not count as a conjunction fallacy.

Now consider this pattern: 50, 50, 50. The estimate of the conjunction is not greater than the estimate of either component. It is equal to one component, and the other

© 2022 Arkes HR et al. *JAMA Network Open*.

component is less than 100%. Therefore, it is scored as the commission of a single conjunction fallacy.

© 2022 Arkes HR et al. *JAMA Network Open*.

eMethods 2. URLs for Preregistration of Substudies

The 3 studies had separate pre-registrations at aspredicted.org

Study 1: #62356

Study 2: #76369

Study 3: #64836

eMethods 3. Response Rate Information

In our 3 studies 285, 496, and 341 invitations were issued to potential respondents respectively. The 3 survey websites were accessed by 73, 92, and 78 respondents respectively. As mentioned in our method section, we did not collect data from a physician in the first study because that person did not attend deliveries. Thus 72 eligible people and 1 ineligible person accessed the website of the first study. The final number of eligible respondents in the 3 studies was 67, 84, and 64 respectively, for a response rate of 89%.

The reason the response rate was extremely high is that all participating physicians were in a panel administered by Reckner Healthcare for the purpose of responding to surveys. Thus, all participants had already volunteered to do such surveys for renumeration. After Reckner sent out their standard invitation to the appropriate group (obstetricians or pulmonologists), all eligible physicians in that group could have responded. However, we stopped enrollment before many of the panel members could respond. How many of the remaining non-enrollees wanted to respond is unknown. Thus, we cannot use as the denominator to calculate response rate the number of respondents who could have taken the survey, because we stopped access to the survey once our target census was reached.

We have no reason to believe that our responders are unrepresentative, but whether the incidence of the conjunction fallacy we found is representative of a broad selection of physicians will require confirmation in future studies. However, the large proportion of physicians committing the fallacy in our study suggests that even if the true proportion is smaller, it will still be a prevalent error.

© 2022 Arkes HR et al. *JAMA Network Open*.

eMethods 4. Survey Guidelines

A reviewer asked us to provide a checklist pertaining to our use of survey guidelines. We used the "CHERRIES" guidelines checklist, which we present on the next page. At the top of the checklist are the following: the bibliographic information of the checklist, a link to the original publication, and the copyright and license information.

We begin with an example of the notice sent to prospective respondents. (The notices for the 3 studies varied in miniscule ways.) This is our response to item #7 in the checklist. Then we present the checklist. Then we present the consent form that contains some of the information requested in the checklist.

Notice sent to potential respondents:

The healthcare industry welcomes your opinions, and we therefore invite you to participate in this upcoming research study:

Compensation: $30 to be redeemed via a Visa® or Amazon® Reward card.

Length: 10 minutes

Topic: Medical Decision Making

Study Reference: Reckner's project number

As with all marketing research, there are a few questions we will ask before you start the survey to ensure that you meet the criteria for the study.

Reckner Healthcare is a leader in medical market research since 1991. We work with healthcare professionals to gather their expert opinions on a range of healthcare developments via online, phone, and in-person studies. Last year alone, we worked with 19,000 healthcare professionals and paid nearly $8 million in honoraria. More info is available at http://healthcaresurveys.reckner.com.

Thank you.

Improving the Quality of Web Surveys: the Checklist for Reporting Results of Internet E-Surveys (CHERRIES)

This checklist has been adapted from the original for *Obstetrics & Gynecology*. Source: Eysenbach G. Improving the quality of Web surveys: the Checklist for Reporting Results of Internet E-Surveys (CHERRIES). J Med Internet Res. 2004 Sep 29;6(3):e34 [erratum in J Med Internet Res. 2012; 14(1): e8.]. Article available at http://dx.doi.org/10.2196/jmir.6.3.e34; erratum available at http://dx.doi.org/10.2196/jmir.2042. Copyright ©Gunther Eysenbach. Originally published in the *Journal of Medical Internet Research* (http://www.jmir.org), 29.9.2004 and 04.01.2012. This is an open-access article distributed under the terms of the Creative Commons Attribution License (http://creativecommons.org/licenses/by/2.0/), which permits unrestricted use, distribution, and reproduction in any medium, provided the original work, first published in the *Journal of Medical Internet Research*, is properly cited. The complete bibliographic information, a link to the original publication on http://www.jmir.org/, as well as this copyright and license information must be included.

Checklist Item	Page Number
Who is the target population? Did you use a convenience sample or some other sampling technique?	7
Which IRB approved this study? If it was exempt, tell us why.	7
What was the informed consent process for participants?	7
Were the participants told the length of time of the survey, data storage process/policies, security of data, name of the investigator, and purpose of study?	e Method 3
Were all visitors to site invited to participate or was this a closed survey?	7
How were potential participants contacted about the survey?<br>Contact by physical or electronic mail?<br>Contact on the site by banner ad?<br>Offline media (i.e. newspapers)?<br>List serves or mailing list?<br>On the site only?	7
Please provide the wording of the advertisement as supplemental digital content	
How were responses to the survey captured?<br>Manually into a database or automatically?<br>If manually, was there a validation process to confirm accuracy?	7
Was completion of the survey mandatory for all who visited the site or voluntary? NO	
In what time frame was the survey offered?	3
Were items in the survey randomized or alternated for different survey respondents? NO	
Did you use adaptive questioning processes?<br>Where certain questions only displayed based on responses to other questions? NO	
Report the completeness checks process?<br>In other words, can you tell how many people started but did not complete the survey? If so, please report this.	7
Did all responses include a non-response option, such as "not applicable" or "rather not say" in order to avoid missing data? NO	
How did you determine unique respondents? Reckner did this<br>Did you use cookies? NO<br>Did you collect IP addresses of client computers?	
How many unique visits to the first page of the survey were there?	7
How many unique visits to the site during the time frame of the survey were there?	7
What was the recruitment rate? Please provide the number of people who filled in the first page of the survey divided by the visitors to the first page of the survey	7
What was the completion rate? Please provide the number of people submitting the last questionnaire page divided by number who agreed to participate or who visited the first page of the survey.	7
How did you handle incomplete questionnaires?	7
How did you assess for a non-representative sample? (propensity scores, weighting of items) NO	

The Ohio State University Consent to Participate in Research

**Study Title:**	**Medical Decision Making**
**Protocol Number:**	**2021E0397**
**Researcher:**	**Hal R. Arkes**

This is a consent form for research participation. It contains important information about this study and what to expect if you decide to participate.

Your participation is voluntary.

Please consider the information carefully. Feel free to ask questions before making your decision whether or not to participate.

Purpose:
Learn how medical experts make decisions.

Procedures/Tasks:
You will make a series of probability estimates about an obstetric situation.

Duration: Today's task will take approximately 10 minutes.

You may leave the study at any time. If you decide to stop participating in the study, there will be no penalty to you. Your decision will not affect your future relationship with The Ohio State University.

Risks and Benefits: The risks of this study are not greater than what you encounter in everyday life. There are no direct benefits to you as an individual, but your responses will help us learn more about how experts make decisions.

Confidentiality:

We will work to make sure that no one sees your online responses without approval. But, because we are using the Internet, there is a chance that someone could access your online responses without anyone's permission. In some cases, this information could be used to identify you.

© 2022 Arkes HR et al. *JAMA Network Open*.

Also, there may be circumstances where this information must be released. For example, personal information regarding your participation in this study may be disclosed if required by state law. Also, your records may be reviewed by the following groups (as applicable to the research):

- Office for Human Research Protections or other federal, state, or international regulatory agencies;
- The Ohio State University Institutional Review Board or Office of Responsible Research Practices

Future Research: Your de-identified information may be used or shared with other researchers without your additional informed consent.

Incentives: For your participation today, you will be paid $30 via Reckner Healthcare.

By law, payments to participants are considered taxable income.

Participant Rights:

You may refuse to participate in this study without penalty. If you choose to participate in the study, you may discontinue participation at any time without penalty. By agreeing to participate, you do not give up any personal legal rights you may have as a participant in this study.

This study has been determined to be exempt from IRB review.

Contacts and Questions:
For questions, concerns, or complaints about the study, or you feel you have been harmed as a result of study participation, you may contact Dr. Hal R. Arkes (arkes.1@osu.edu).

For questions about your rights as a participant in this study or to discuss other study-related concerns or complaints with someone who is not part of the research team, you may contact the Office of Responsible Research Practices at 1-800-678-6251 or hsconcerns@osu.edu.

Providing consent

I have read this page and I am aware that I am being asked to participate in a research study. I have had the opportunity to ask questions by contacting Dr. Arkes and have had them answered to my satisfaction. I voluntarily agree to participate in this study. I am not giving up any legal rights by agreeing to participate.

To print or save a copy of this page, select the print button on your web browser.

Please click the button below to proceed and participate in this study. If you do not wish to participate, please close out your browser window.

© 2022 Arkes HR et al. *JAMA Network Open*.

eAppendix 1. Analysis Comparing the Discrepancy Between the Conjunction Estimate and the Product of the 2 Components Among Physicians Who Committed Any Conjunction Fallacy vs Physicians Who Did Not

In Study 1 (brow presentation) we compared the discrepancy between the conjunction estimate and the product of the 2 components for those who committed some form of the conjunction fallacy and those who did not. The 50 respondents who succumbed to the fallacy had a mean discrepancy of 16.1%. The 17 who did not succumb had a mean discrepancy of 3.3%. This difference was significant [$t(58.4) = 5.23$, $P < .001$], Cohen's $d = 1.06$, (95%CI = .47 – 1.63). Not committing the conjunction fallacy was associated with a reduction of the discrepancy between one's estimate of the conjunction and the product of the 2 components.

In Study 2 (pulmonary nodule) we compared the discrepancy between the conjunction estimate and the product of the 2 components for those who committed some form of the conjunction fallacy and those who did not. The 73 respondents who succumbed to the fallacy had a mean discrepancy of 22.0%. The 11 who did not succumb had a mean discrepancy of 5.4%. This difference was significant [$t(53.1) = 7.74$, $P < .001$], Cohen's $d = 1.21$, (95%CI = .54 – 1.86). Not committing the conjunction fallacy was again associated with a reduction of the discrepancy between one's estimate of the conjunction and the product of the 2 components.

In Study 3 (debiasing brow presentation) We compared the discrepancy between the conjunction estimate and the product of the 2 components for those who committed some form of the conjunction fallacy and those who did not. The 45 respondents who succumbed to the fallacy had a mean discrepancy of 25.4%. The 19 who did not succumb had a mean discrepancy of only 0.4%. This difference was significant [$t(62) = 6.44$, $P < .001$], Cohen's $d = 1.76$, (95%CI = 1.14 – 2.37). Not committing the conjunction fallacy was again associated with a reduction of the discrepancy between one's estimate of the conjunction and the product of the 2 components.

© 2022 Arkes HR et al. *JAMA Network Open*.

We have added these analyses to show that not committing the conjunction fallacy results in a substantial improvement in the correspondence between one's conjunctive estimate and the product of the 2 components' estimates. The over-estimation of the conjunction relative to the product is reduced when the conjunction fallacy is absent.

© 2022 Arkes HR et al. *JAMA Network Open*.

eAppendix 2. Further Analysis of the Frequency of the Commission of the Single Conjunction Fallacy in the 3 Substudies

A reviewer asked us to consider how often the commission of the conjunctive fallacy was due to the relation between the conjunctive estimate and the estimate of the first component and how often the commission of the conjunctive fallacy was due to the relation between the conjunctive estimate and the estimate of the second component. In the following chart we omit those instances in which a double conjunction fallacy occurred, because in those instances the conjunctive estimate was greater than both components.

	First Component	Second Component
Study 1	44	1
Study 2	27	27
Study 3	39	3

In Studies 1 and 3 the conjunction exceeded the first component far more often than the second component. This was not the case in Study 2. What might account for this difference? Our conjecture is the following. In Studies 1 and 3 there are two steps to consider, namely, conversion to OP and vaginal delivery. In Study 2 the first component represents a base rate, namely, the pretest probability that the patient has a malignancy. In some of their earliest judgment/decision making research, Kahneman and Tversky[1] showed that base rate information is underutilized. If "underutilized" implies "underestimated," then one might expect the conjunctive estimate would be less likely to fall beneath the low estimate of the first conjunct, and single conjunction errors would be plentiful. However, we assert that Kahneman and Tversky did not mean that the magnitudes of base rates were underestimated. Furthermore Morgan et al.[2] presented evidence that pre-test probabilities are usually overestimated, not underestimated. The research by Kahneman and Tversky clearly shows that "underutilized"

© 2022 Arkes HR et al. *JAMA Network Open*.

means “insufficiently considered.” If persons in Study 2 insufficiently considered the base rate (Component 1), then strong propensity of physicians to assign a conjunction estimate higher than a component exhibited in Studies 1 and 3 would be more likely to occur to Component 2, which would attract relatively more attention than it did in the other 2 studies in which the first component was not an insufficiently regarded base rate. This would result in the conjunction being more likely to exceed only Component 2 in Study 2 than in the other 2 studies. This is the result we obtained.

© 2022 Arkes HR et al. *JAMA Network Open*.

eFigure 1. Probability Estimates From Participants in Substudy 1 (Brow Presentation During Labor)

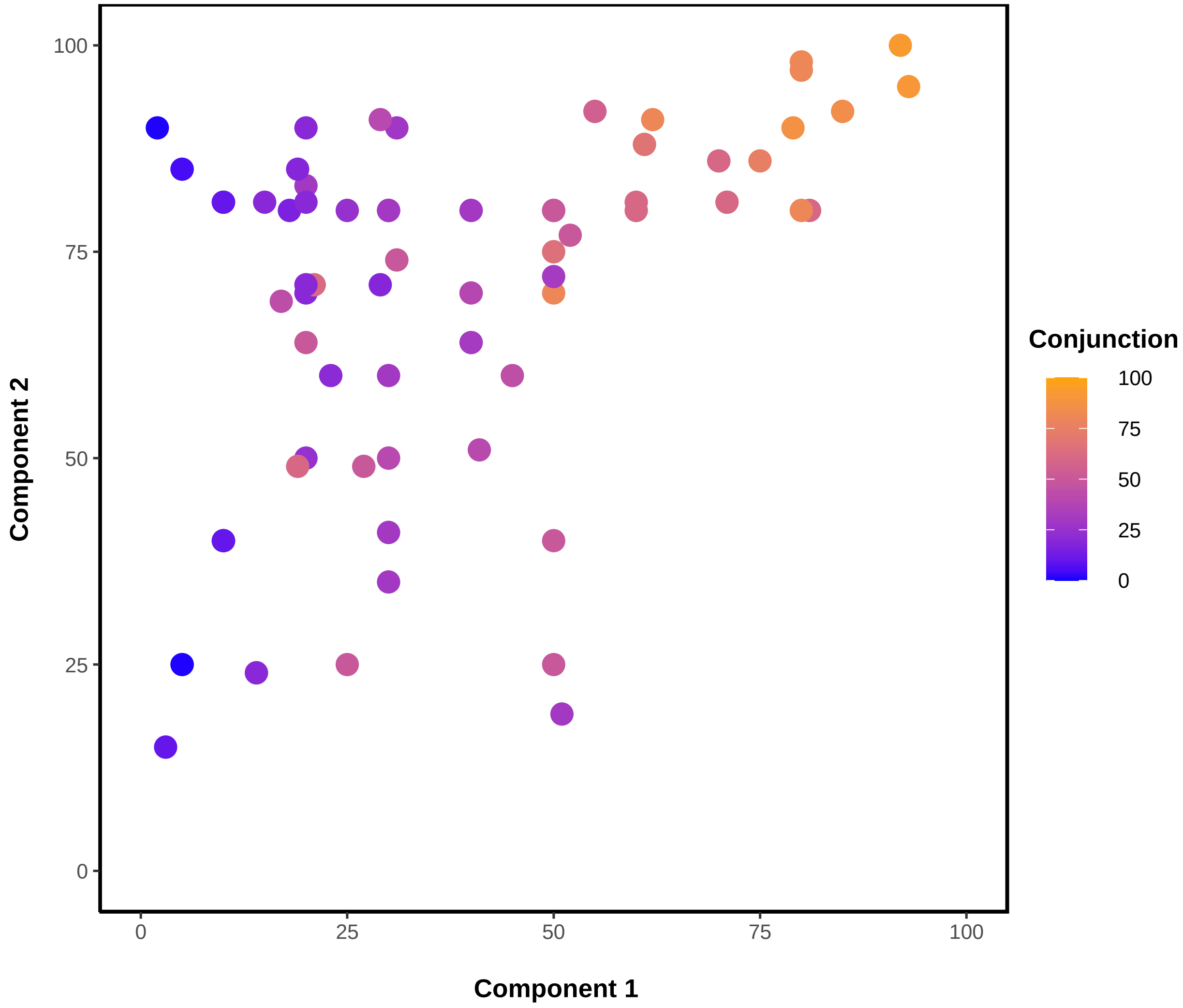

All respondents' probability estimates for the first component (horizontal axis), the second component (vertical axis), and the overall conjunction (color of each data point).

© 2022 Arkes HR et al. *JAMA Network Open*.

eFigure 2. Probability Estimates From Participants in Substudy 2 (Pulmonary Nodule)

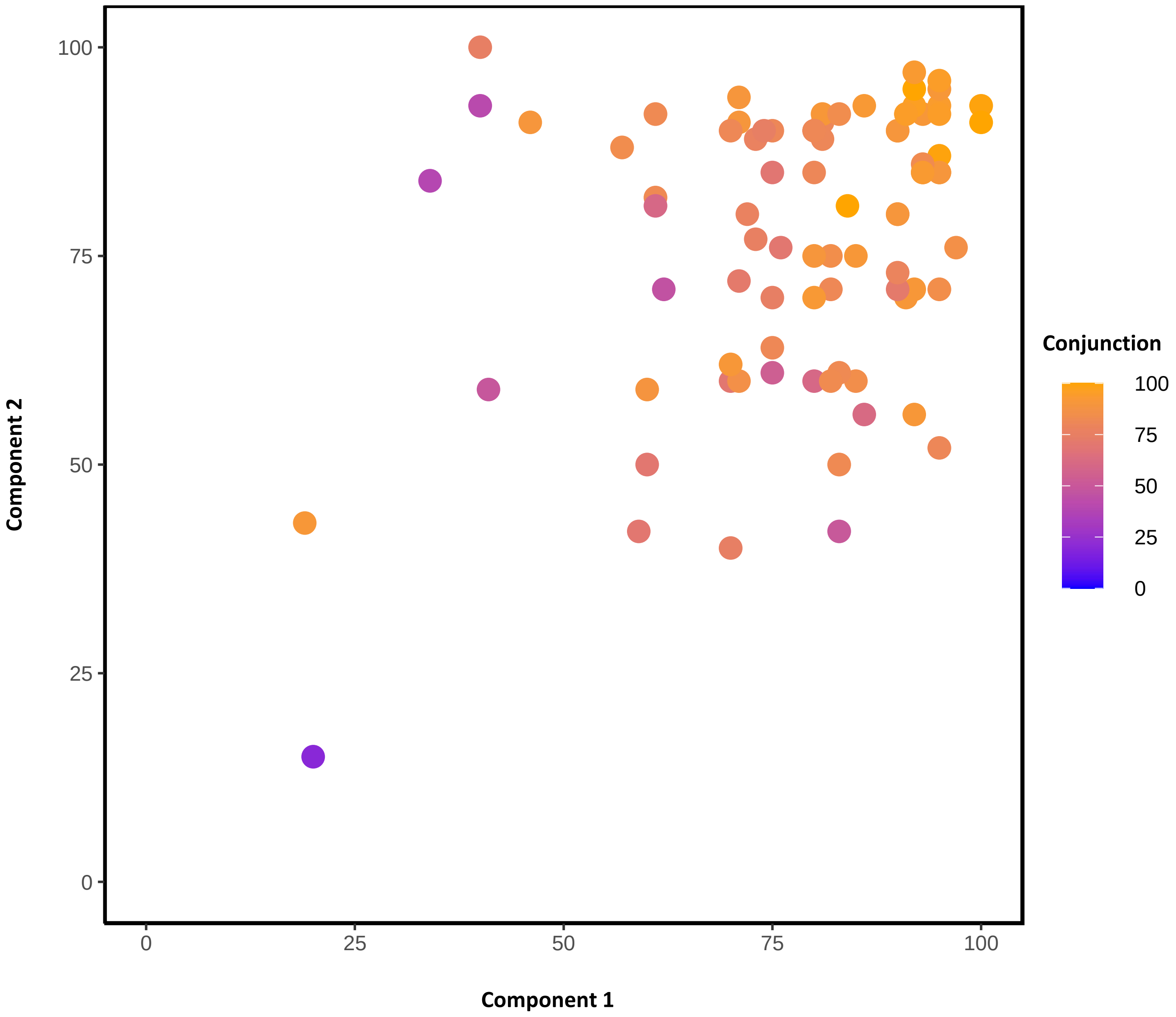

All respondents' probability estimates for the first component (horizontal axis), the second component (vertical axis), and the overall conjunction (color of each data point).

© 2022 Arkes HR et al. *JAMA Network Open*.

eFigure 3. Probability Estimates From Participants in Substudy 3 (Debiasing Brow Presentation During Labor)

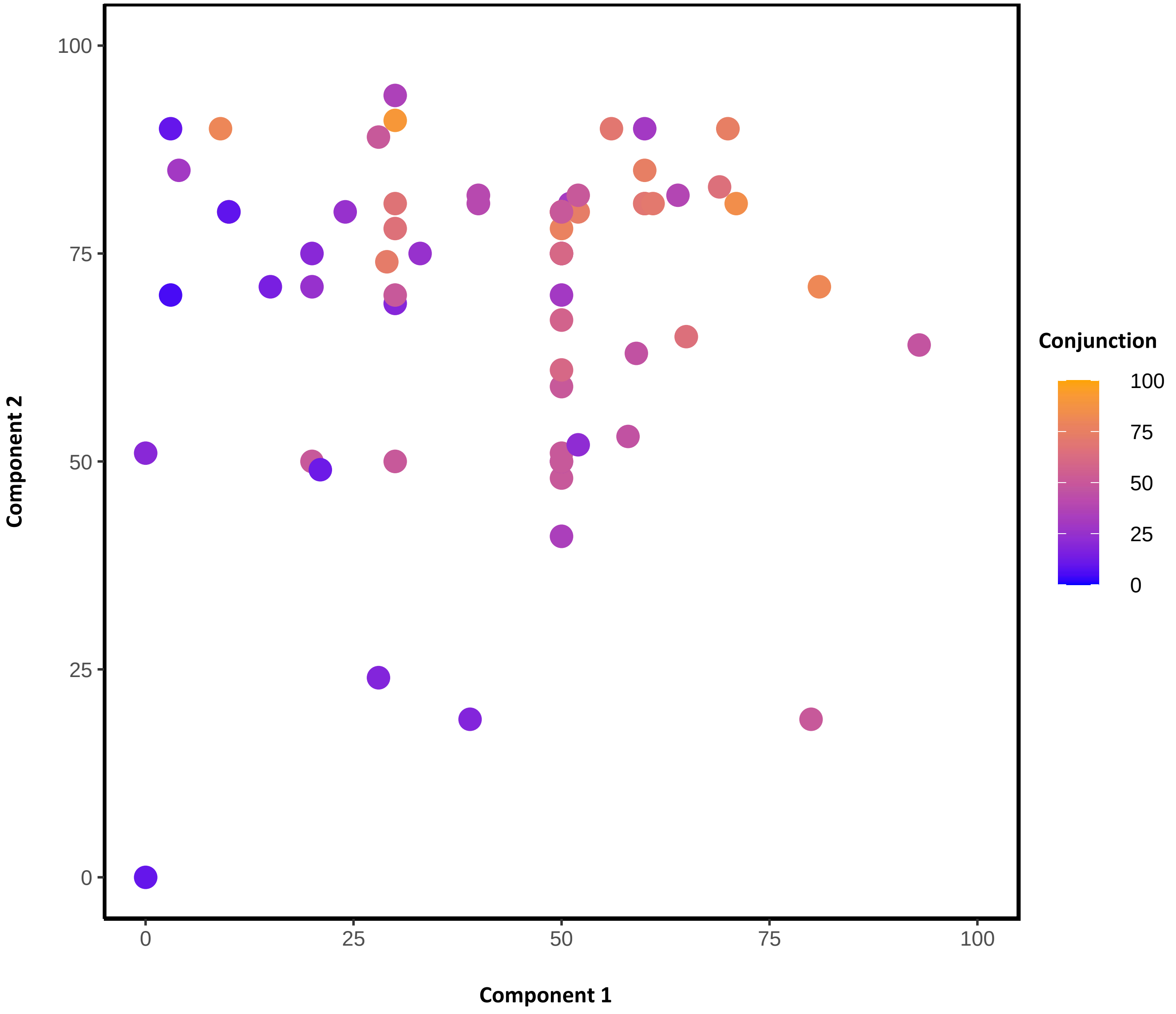

All respondents' probability estimates for the first component (horizontal axis), the second component (vertical axis), and the overall conjunction (color of each data point).

© 2022 Arkes HR et al. *JAMA Network Open*.

eReferences

1. Kahneman, D, Tversky, A. On the psychology of prediction. *Psychol Rev*.1973;80:237-251.
2. Morgan DJ, Pineles L, Owczarzak J, et al. Accuracy of practitioner estimates of probability of diagnosis before and after testing. *JAMA Intern Med*.2021;181(6):747-755. doi:10.1001/jamainternmed.2021.0269

© 2022 Arkes HR et al. *JAMA Network Open*.
